# Sugar, mineral, B-vitamins profiles and radical scavenging activity of Royal jelly collected at different harvesting times

**DOI:** 10.1371/journal.pone.0347342

**Published:** 2026-04-21

**Authors:** Eyuel Welelaw, Abera Belay, Tadele Alemu, Teferi Damto, Admasu Addi, Mesfin Getachew Tadesse, Endale Amare, Feriehiwote Weldeyohanis Gebremariam, Ibrahim Ahmed Sherif, Henock Woldemichael Woldemariam

**Affiliations:** 1 Wachemo University, College of Agricultural Science, Department of Food Science and Post-Harvest Technology, Hossana, Ethiopia; 2 Addis Ababa Science and Technology University, Department of Food Science and Applied Nutrition, Addis Ababa, Ethiopia; 3 Addis Ababa Science and Technology University, Biotechnology and Bioprocess Center of Excellence, Addis Ababa, Ethiopia; 4 Oromia Agricultural Research Institute, Holeta Bee Research Center, Holeta, Ethiopia; 5 Addis Ababa Science and Technology University, Department of Industrial Chemistry, Addis Ababa, Ethiopia; 6 Nutrition, Environmental Health and Non-Communicable Diseases Research Directorate, Ethiopian Public Health Institute, Addis Ababa, Ethiopia; 7 Addis Ababa Science and Technology University, Department of Chemical Engineering, Addis Ababa, Ethiopia; University of Veterinary and Animal Sciences, PAKISTAN

## Abstract

Royal jelly is produced by young worker honeybees that support the growth of their larvae. Human beings can harvest and use it as functional food due to its high content of nutrients and beneficial human health effects. This study aims to investigate how harvesting time affects sugar and minerals, B vitamins profiles, and antioxidant (free radical scavenging) activity. Royal jelly samples were collected at third and sixth days of secretion and deposition by honeybees *(Apis mellifera*) in Holeta Bee Research Center, Ethiopia. Sugar, minerals and B-vitamins profiles and radical scavenging potential were examined by using standard methods. The highest sugar level was fructose (0.48 ± 0.03%) and the lowest was maltose (0.05 ± 0.01%) harvested on the third and sixth days, respectively and significant difference (ρ < 0.05) among fructose and sucrose. The minerals concentrations in descending order are K (4144.32 ± 174.98 mg/kg)> Mg (309.61 ± 8.33 mg/kg)> Ca (235.26 ± 1.05 mg/kg)> Na (155.60 ± 1.76 mg/kg) and the most abundant elements in royal jelly and significantly vary (ρ < 0.05) based on harvesting time, except in Mg. The highest vitamin content was vitamin B9 (14.8 ± 0.07 mg/kg) and the lowest was vitamin B3 (0.57 ± 0.06 mg/kg) harvested on third and sixth days, respectively and significant difference (ρ < 0.05). Royal jelly exhibited the strongest free radical scavenging activity (75.38%) and the lowest (59.97%), collected from the third and sixth days respectively and significant difference (ρ < 0.05). The inhibition concentration (IC_50_) value of royal jelly collected on third and sixth was 4.84% and 6.56%, respectively. Thus, the evaluated nutritional components and antioxidant properties in the royal jelly altered through harvesting times, and varied between royal jelly collected at different times, and found that royal jelly collected at third days more nutritious.

## Introduction

Royal jelly (RJ) is a nutritious functional food produced by the young nurse bees’ hypopharyngeal gland. It is essential to both the development of the queen bee and the determination of caste [1]. Royal jelly has been utilized for human health care from ancient times [2]. It remains crucial in traditional and folkloristic medicine today [3]. Royal jelly is also a functional food that might have biologically active ingredients and offer health advantages over and beyond basic nutrition [4].

Royal jelly is known for its rich nutritional profiles and potential health benefits [5]. Due to the abundance of remarkable proteins, lipids, sugars, vitamins, hormones, enzymes, mineral substances, and specific essential elements that function as biocatalysts in the human body’s cell regeneration processes, this rich concentrated food is beneficial for both humans and bees [6].

Royal jelly’s unique combination of sugars, minerals, and B vitamins helps to promote the health of the skin and nervous system, increase immunity, and improve energy levels [7]. For royal jelly, sugars are among the main components, and their quantification is of evident interest for its quality. Studies on sugar typically compare the contents of the three main sugars; fructose, glucose, and sometimes sucrose [8]. Royal jelly contains several essential minerals that support various bodily functions [3,9]. It is also rich in several B-vitamins and other vitamins, which are important for energy production, brain function, and overall cellular health [10]. These merits of royal jelly vary depending on seasonality, geography, among different bee races, types of feed and levels, harvest time, and larval age at grafting time [11–14].

Different researchers have reported on the nutritional composition of royal jelly. For example the reports regarding Khalfan Saeed Alwali Alkindi, El–Keblawy [15] and Oršolić and Jazvinšćak Jembrek [5] indicated that royal jelly’s chemical composition, especially its sugar content, varies greatly depending on the plant and bee species, harvest season, collection technique, and the availability of forage sources in the environment that are compatible with the bees’ natural diets, such as pollen and nectar. But the effect of harvesting time on royal jelly and data available in the literature are highly variable.

There is an increasing interest in royal jelly due to the possible benefits in medicine, nutrition, and cosmetics, which has led to significant investment in research and development [15]. Natural antioxidants have recently drawn interest from researchers and scientists due to their potential to protect human health by preventing a number of chronic diseases brought on by free radical damage [16].

Antioxidant properties have been demonstrated for royal jelly [17,18], and on the basis of its nutritional worth (vitamins, polyphenolic compounds, or protein fractions) a growing number of commercial royal jelly products in different forms are available on international markets. However, the antioxidant properties of royal jelly at different times of harvest need further investigation.

Sugar composition, minerals, and vitamin profiles are the most widely used criteria for evaluating the quality of royal jelly and possess high nutritional value [19]. However, no previous investigation has been done on the harvesting time effect on royal jelly’s chemical composition and potential health benefits in relation to radical scavenging activity royal jelly. Therefore, the current study aimed to investigate the effects of harvesting time on the sugar and mineral content, B-vitamin profiles, and free radical scavenging activity of royal jelly.

## Materials and methods

### Research design

To examine the sugar and minerals, B- vitamins profiles, and antioxidant (free radical scavenging) activity of royal jelly, it was laid out in a complete randomized design (CRD). It has one factor (harvesting time of royal jelly) and two levels (T1-72 hours and T2-144 hours) based on the modified method of [20].


 Yi = μ + HTi + εi 

Where:

Yi = The overall observations of the i^th^ treatment

μ = Population mean

HTi = Treatment effect of the i^th^ harvesting time

εi = The error term of the i^th^ treatment and replication

### Sample collection

Samples of royal jelly were collected at Holeta Bee Research Center. The Holeta Bee Research Center (HBRC) is located in Holeta town, Ethiopia. It is around 35 kilometers west of Addis Ababa at about 9°30′ North latitude and 38°30′ East longitude. The center is situated in Ethiopia’s central highlands. It is situated between 2,250 and 2,500 meters above sea level. The average yearly temperature was 23.5 °C at its highest point and 7.3 °C at its lowest. The surrounding scenery is typical of the Ethiopian plains, with gently sloping hills and sporadic hilly areas. The ecological parameters in this key area are ideal for bee research and development.

Royal jelly production and harvesting in the apiaries followed standard protocols [21]. This procedure describes how to produce and harvest royal jelly, from selecting colonies to storing them and ensuring the best quality. The selected colonies are built by providing supplementary feed during the dearth period (July and August), which is designed to support royal jelly production. During the collection of royal jelly samples (from October to December), the experimental colonies totally depend on naturally existed honey plants (*Guizotia scabra,* *Trifolium spp., Brassica napus, Bidens spp., Plantago lanceolatum,* and *Rumex nervosus)* that honeybee feed to become strong colony and did not give any treatments. Honeybees visit flowers to obtain pollen and nectar during the royal jelly gathering time. Honeybees require and consume pollen and nectar in order to build robust colonies, which help us to collect royal jelly. Royal jelly is secreted by the worker bee’s gland. However, at dearth period, due to the absence of flowers before the time of royal jelly collection, the selected colonies given supplementary feed to build the colony and facilitate the production of royal jelly. This dearth period feed typically includes honey or sugar syrup. The production of royal jelly begins with the meticulous selection of healthy and productive colonies of honey bees (*Apis mellifera*). The production of royal jelly depends on building colonies during the dearth period, and these colonies have sufficient number of nurse bees. Ten productive colonies of *Apis mellifera* honeybees were thus selected randomly and kept in a queen rearing place, at the Holeta Bee Research Center apiary. In order to ensure the best possible health and productivity, these experimental colonies were carefully maintained under closely controlled circumstances. Using Doolittle grafting queen rearing technique, which comprised placing young larvae into plastic queen cell cups to induce royal jelly secretion by nurse bees, royal jelly was extracted from the selected colonies. To evaluate the impact of harvesting time on the composition and the royal jelly’s bioactivity, the collection was planned to conduct at three distinct intervals, namely on the third, sixth and ninth days following grafting. However, by the ninth day, the natural production of royal jelly in the queen cells had significantly declined, making it no longer possible to collect any viable samples at this stage. To maintain the nutritional and biochemical integrity of the extracted royal jelly until laboratory examination, it was carefully taken from the queen cells using sterile instruments and promptly kept at −80 °C.

### Grafting of young larvae

For Doolittle grafting technique, among ten established experimental colonies, a total of 5 productive strong honeybee colonies (two breeders, and three starters) with uniform strength and similar species (*Apis mellifera*) in Langstroth hive with the population size of first super were selected, based on the criteria utilized for queen rearing. There was no variation on the royal jelly collection among colonies. The starter colonies were prepared 24 hours before grafting by removing combs containing eggs, young larvae, and queens. For each starter colony, a grafting frame consisting of two bars holding 20 artificial queen cell cups was set up. Then, in the laboratory, young larvae were grafted into each queen cell cup that consisted a drop of a 1:1 (water-to-royal jelly) solution. These grafted larvae were then immediately introduced into the prepared starter colonies for royal jelly collection at various harvesting intervals.

### Harvesting

The royal jelly-filled cups were carefully harvested from the hive after a specific timeframe, usually between 72 and 144 hours. Royal jelly samples for this investigation were taken at 72, 144, and 216 hours. However, at 216 hours or nine days, no royal jelly was collected, and the natural production of royal jelly in the queen cells had significantly declined, making it no longer possible to collect any viable samples at this stage. Royal jelly samples were collected from October to December, at the mean temperature of 20 °C. For the royal jelly to be pure and of high quality, this procedure of sample collection is followed precisely.

### Storage

After being harvested, the cups filled with royal jelly are kept at −80 °C to preserve their quality and freshness until further analysis.

### Determination of sugars, minerals, B-vitamin profiles, and antioxidant (free radical scavenging) activity of royal jelly

#### Mineral profile analysis.

Mineral analysis in royal jelly was evaluated using standard procedure methods [22]. The mineral components in royal jelly samples were determined by Atomic Emission Spectroscopy using the Agilent Technologies (4200MPAES) Microplasma Atomic Emission Spectrometer with MP Expert (Demo Mode). The high temperature requirements were produced using an air-nitrogen mixture. About 0.2 g of the homogenized royal jelly sample was weighed and put directly into the high-pressure digestion vessel PTFE-TFM for the digestion step or process. To each sample, 65% nitric acid (7 mL) and 30% hydrogen peroxide (1 mL) were added. The samples were placed in the Rotor 8XF100 after being sealed in the ceramic covering. Microwaves were used to carry out the digestion. In 145 °C/5 minutes/75% power, 180 °C/10 minutes/90% power, and 100 °C/10 minutes/40% power were the three stages of the microwave digestion program. After being digested, the samples were transferred into 25 mL volumetric flasks and ultrapure water was added until 25 mL was full. Each element’s calibration curves (S6-S12 Figs in S1 File) were performed using standards that were serially diluted from their working standard and had varying concentrations (S3 Table in S1 File). The working standard was prepared from stock solutions.

#### Sugar profile analysis.

Analysis of sugar was done based on method described by [23]. The proposed technique for determination of the main royal jelly sugars was easily applicable in a laboratory that was set up to determine honey sugars and has sufficient sensitivity [23]. Sugar standards; D-(+)-fructose (Sigma Aldrich, Lot 092K0137, EC No: 200-333-3), D-(+)-glucose (Sigma Aldrich, EC No: 20110425), sucrose (Sigma Aldrich, S-8501 Lot 84H0557), and D-(+)-maltose monohydrate (TCI Lot. VW6L-BG, M0037), were prepared using five-level serial dilutions, and the sugars with different modifications were determined using HPLC [24]. High-performance liquid chromatography (HPLC) (HPLC-1260 Infinity Series Agilent Technologies) with a differential refractive index detector was used to determine the sugars profile [25,26]. Two grams (2 g) of royal jelly were taken from a thoroughly homogenized sample and dissolved in a 70:30, v/v ratio of acetonitrile to water. A syringe filter (HSW Henk Sass Wolf GmbH), with a pore size of 0.45 μm was used to filter the solution of each royal jelly sample before it was transferred to autosampler vials for HPLC sugar analysis. The peaks in the HPLC chromatogram (S1-S5 Figs in S1 File), which are used to determine sugars, are found by comparing the retention times obtained from standards. On the crude royal jelly, the results are expressed as a weight-to-weight (w/w) percentage (g/100 g) of each sugar.

### Vitamin profile analysis

The quantification of B- vitamins: riboflavin (B2), niacin (B3), pyridoxine (B6), and folate (B9), was performed by High-Performance Liquid Chromatography (HPLC) (HPLC-1260 Infinity Series Agilent Technologies) following established methodologies [27]. Chromatographic separation was achieved using reversed-phase C₁₈ columns (200 mm × 4.6 mm, 4 µm particle size) with gradient elution using a mobile phase consisting of 10 mM phosphate buffer (pH 3.0) and acetonitrile (15% v/v). For sample preparation of B- vitamins (B2, B3, B6, and B9) 0.2 g royal jelly was weighed. The royal jelly sample was transferred into a 50 mL volumetric flask and 15% of methanol solution was added. To enhance peak resolution, hexane sulfonic acid was incorporated into the mobile phase. The analytical conditions were as follows: column temperature, 40°C; and flow rate ranging from 1.2 mL/min. Prior to analysis, samples underwent rigorous preparation involving homogenization followed by acid hydrolysis (0.1 N H₂SO₄) to extract vitamins from the matrix. Protein precipitation was performed using trichloroacetic acid to eliminate interfering proteins, with subsequent filtration through 0.22 µm syringe filters (HSW Henk Sass Wolf GmbH) to ensure sample purity. Detection was accomplished using a photodiode array detector (UV-DAD), enabling simultaneous multi-wavelength monitoring to optimize sensitivity and selectivity for each vitamin: 270 nm for riboflavin (B2), 260 nm for niacin (B3), 290 nm for pyridoxine (B6) and 280 nm for folate (B9). Standard calibration curve for each vitamin standard confirms a linearity of (R² = 0.995–0.999).

### Antioxidant potential of royal jelly

#### Radical scavenging potential assay.

Using the methods of [28], the scavenging activity of royal jelly samples against the 2,2-Diphenyl-1-picrylhydrazy (DPPH) radical was examined. In the assay mixtures were 2.7 mL of methanolic solution containing DPPH radicals (Sisco Research Laboratories Pvt. Ltd. (SRL), India) (0.024 mg/ml) and 0.5 mL of royal jelly. The mixture shaken on a vortex mixer (Thermo Fisher Scientific, USA), it was incubated in a water bath (Drawell Scientific, Drawell Scientific Co., Ltd., China; Type 1003: 10701986C) at 25 °C for 90 minutes in the dark. The absorbance at 517 nm was then measured against a blank. With the exception of DPPH, all of the reagents were taken as blanks. The standard utilized was ascorbic acid (0–10 mg/mL). The radical scavenging activities of the DPPH radical were expressed as a percentage of inhibition (S4 Table in S1 File.), were calculated using the equation that follows.


$$\;\%\;inhibition=\;\frac{\left(Ab\;blank-Abs\;sample\right)}{Ab\;sample}\times \;\text{1}\text{0}\text{0}$$ (1)

Where Abs sample = sample absorbance at 517 nm, and Abs blank = blank absorbance at 517 nm.

### IC₅₀ determination

The half-maximal inhibitory concentration (IC₅₀) was determined using GraphPad Prism version 10.4.2 (San Diego, USA) based on the modified methods [29,30]. Dose-response data were obtained by plotting royal jelly concentration in percentage (mass per volume or μg/mL) (log₁₀-transformed, X-axis) against the corresponding biological response (percentage inhibition or activity, Y-axis). The data were fitted using a nonlinear regression model based on a three-parameter logistic (sigmoidal) equation:


$$R\text{esponse}=\frac{\text{Maximum response}}{\text{1}+\text{10}\left(\text{logIC50}-\text{X}\right)}$$ (2)

where Y represents the response, X is the log₁₀ concentration of the royal jelly, and IC₅₀ is the concentration producing 50% inhibition. IC₅₀ values were automatically calculated by GraphPad Prism from the fitted curves (S5 Table in S1 File).

### Statistical analysis

The sugar, minerals, vitamins-B profiles and antioxidant properties data was analyzed by SAS, 2002 using a one-way analysis of variance (ANOVA). The Tukey test was performed for the statistical evaluation of the data obtained. Statistical analyses of IC_50_ values were done using Graph Pad Prism (version 10.4.2). Results were reported as Mean ± SD. The level of significance was set at P < 0.05.

## Results and discussion

### Sugar profile of royal jelly

**Fig 1:**
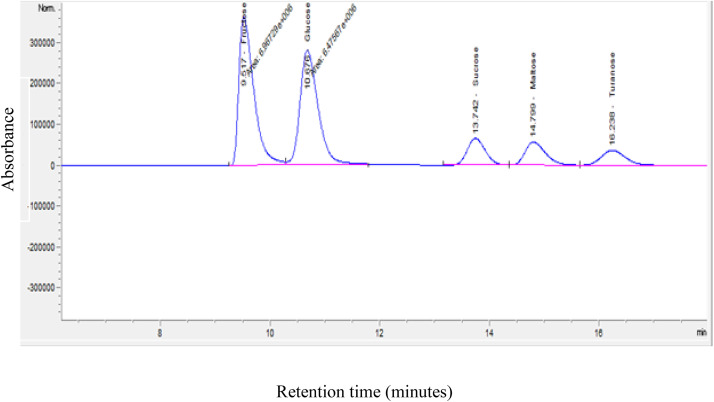
HPLC chromatogram for sugar profile of royal jelly sample.

Determining the sugar content of RJ samples is essential for evaluating their quality. The metabolic pathways of lipids and carbohydrates are significantly influenced by sugars. Sugars may also be employed as a criterion to look for signs of likely adulteration in honey and RJ. As a result, sugars are thought to be a crucial component in determining the quality of RJ. Fructose, glucose, and sucrose are the primary RJ sugars, according to research [31]. Thus, sugar determination is a critical important step in achieving a quality standard. The chromatogram obtained in analyzing a mixt of sugar standards in a royal jelly sample (fructose, glucose, sucrose, and maltose) was shown in Fig 1. For the sugars profile of the analysed royal jelly sample, the HPLC chromatogram found retention times of Fructose = 9.517, Glucose = 10.676, Sucrose = 13.742, and Maltose 14.799 minutes, as presented in Fig 1. The sample’s sugar content was calculated by comparing the corresponding peak areas to those of the pure standard solutions (S1 Table in S1 File).

Table 1 illustrates the mean ± SD percentage values for sugar profiles in the samples of royal jelly collected on the 3^rd^ and 6^th^ days. The mean ± SD values of glucose and maltose in royal jelly were (0.36 ± 0.08 and 0.12 ± 0.06%) and (0.15 ± 0.03 and 0.05 ± 0.01%), respectively (Table 1). There were no significant differences (p > 0.05) observed between the two royal jelly samples for sugar profiles (S7 and S9 Tables in S1 File). The results suggest that the concentrations of glucose and maltose in royal jelly do not vary significantly across the third and sixth days harvesting times. This implies that these two sugars remain relatively stable regardless of when the royal jelly is collected and this is in agreement with previous reports of [32,33] A sample of royal jelly collected on the 3^rd^ (0.15 ± 0.03%) and 6^th^ (0.05 ± 0.01%) days indicates a low maltose content, which could imply good quality and is in line with other studies where samples in royal jelly 1, royal jelly 5, and royal jelly 6, the amounts of maltose are 0.19%, 0.08%, and 0.12%, respectively [34].

**Table 1:** Sugar profile (mean ± SD) of royal jelly collected on the 3^rd^ and 6^th^ days (%).

Treatments	Fructose	Glucose	Sucrose	Maltose
Royal jelly 3^rd^ day	0.48 ± 0.03^a^	0.36 ± 0.08^a^	0.24 ± 0.03^a^	0.15 ± 0.03^a^
Royal jelly 6^th^ day	0.16 ± 0.03^b^	0.12 ± 0.06^a^	0.08 ± 0.03^b^	0.05 ± 0.01^a^

SD-standard deviation; Values represent the average of duplicates ± standard deviation; Mean values in the same column indicated with different letters are statistically significant different (ρ < 0.05).

A significant difference (ρ < 0.05) was observed among the fructose and sucrose in royal jelly samples (S6 and S8 Tables in S1 File). The fructose content for third and six days royal jelly was 0.48 ± 0.03% and 0.16 ± 0.03%, respectively. Kanelis, Liolios [35] reported that, royal jelly collected after 72 hours or the three-day grafting period had high fructose (3%), which was agreement with the current royal jelly investigation. The sucrose content for third and six days royal jelly was 0.24 ± 0.03% and 0.08 ± 0.03%, respectively. Based on the available literature, the range of fructose (3–13%) and sucrose (0.5–2%) contents in royal jelly is given [34]. While the fructose content varies, the average sucrose content is in line with the literature.

According to Zheng, Hu [32], the nutritional components in royal jelly depended on the harvest time. Glucose and fructose are the main sugars in royal jelly, and the high fructose level in regard to glucose and other sugars may be an indictors of the bees’ particular diet and nectar quality [36]. Sabatini, Marcazzan [36] reported that, their contents in royal jelly gradually increased over time. However, the results in this study for 6 days were lower than the values of royal jelly collected in 3 days. Therefore, sugar content of royal jelly decreased as harvesting time delaying in the current study and this is supported by Zheng, Hu [32] that report fresh royal jelly’s sugar concentration decreased on the first day. According to Mokaya, Njeru [18], in contrast to honey, which is produced directly from floral nectar or plant exudates, royal jelly is released by nurse bees after partially consuming honey and pollen, which may explain why it has lower sugar levels during different harvest times. The low sugars in royal jelly could be due to the way it is produced and effects of harvesting times, i.e., secreted by nurse bees after partial consumption of honey and pollen, unlike honey which is directly processed from floral nectar or plant exudates [32]. Nevertheless, sugars are necessary for promoting and controlling metabolic processes, they play an important role in the biochemical and physiological aspects of all living organisms [31]. The decrease in sugar content of royal jelly as harvesting time is delayed can be attributed to several factors related to bee physiology, hive dynamics, and the natural production process of royal jelly. Royal jelly produced by nurse bees to feed young larvae (especially future queens). As larvae grow, they consume more royal jelly, reducing the residual sugar content in harvested royal jelly if collection is delayed [32]. Fresh royal jelly also contains enzymes like glucose oxidase that are able to hydrolyze sugars (like glucose and fructose) slowly over time. Thus, delayed harvesting may cause partial hydrolysis of sugars, reducing their content [36,37].

### Minerals profile of royal jelly

The mean concentrations (mg/kg) of mineral profiles for royal jelly collected on the 3^rd^ and 6^th^ days were presented in Table 2. It was found that Ca has 235.26 ± 1.05 mg/kg on the 3^rd^ day and 192.94 ± 8.10 mg/kg on the 6^th^ day; Fe was 17.78 ± 0.39 mg/kg on the 3rd day and 9.48 ± 0.453 mg/kg on the 6th day; Zn was 24.79 ± 1.68 mg/kg on the 3^rd^ day and 20.25 ± 1.344 mg/kg on the 6^th^ day; Na was 155.60 ± 1.76 mg/kg on the 3rd day and 120.49 ± 4.23 mg/kg on the 6th day; Mn was 1.6700 ± 0.06 mg/kg on the 3rd day and 1.0650 ± 0.08 mg/kg on the 6^th^ day; K was 4144.32 ± 174.98 mg/kg on the 3^rd^ day and 3245.12 ± 89.78 mg/kg on the 6^th^ day; and Mg was 309.61 ± 8.33 mg/kg on the 3^rd^ day and 304.72 ± 3.52 mg/kg on the 6^th^ day. The mineral profiles of royal jelly significantly vary (p < 0.05) based on harvesting time, except in Mg (p > 0.05) (S11-S17 Tables in S1 File). This is due to the fact that more macro and trace elements are needed by younger larvae than by older ones. However, the difference in Mg content between different harvest times, like 3^rd^ and 6^th^ days, might not be as significant as the changes observed in other minerals content. Biological, physiological, and environmental variables all contribute to the relatively constant magnesium (Mg) concentration of royal jelly during various harvesting seasons [38] and this agreed with the current study.

**Table 2:** Minerals profile of royal jelly collected on the 3^rd^ and 6^th^ days (mg/kg).

Treatments	Ca	Fe	Zn	Na	Mn	K	Mg
Royal jelly 3^rd^ day	235.26 ± 1.05^a^	17.78 ± 0.39^a^	24.79 ± 1.68^a^	155.60 ± 1.76^a^	1.6700 ± 0.06^a^	4144.32 ± 174.98^a^	309.61 ± 8.33^a^
Royal jelly 6^th^ day	192.94 ± 8.10^b^	9.48 ± 0.453^b^	20.25 ± 1.34^b^	120.49 ± 4.23^b^	1.0650 ± 0.08^b^	3245.12 ± 89.78^b^	304.72 ± 3.52^a^

SD-stands for standard deviation; Values represent the average of duplicates ± standard deviation; Mean values in the same column marked with different letters are statistically significant different (ρ < 0.05).

The highest concentration of K (4144.32 ± 174.98 mg/kg) was obtained from royal jelly harvested on the 3^rd^ day. Stocker, Schramel [38] reported that the highest concentration of K (3050 mg/kg) was present in royal jelly harvested in July after grafting and this is lower than the current study’s results.

Due to the larvae’s developmental requirements, the 3-day is the peak time when royal jelly has the maximum K levels [39]. The lowest concentrations of Mn (1.0650 ± 0.08 mg/kg) were present in royal jelly harvested at 6^th^ days after grafting (Table 2). Relatively, similar Mn concentrations were reported in royal jelly and it is the only element with smaller element concentrations (1.06 mg/kg) [38]. The most abundant mineral among those examined was potassium, which was followed by magnesium, calcium, and sodium. Their potassium, magnesium, and calcium levels were measured at 4144.32 ± 174.98 mg/kg, 309.61 ± 8.33 mg/kg, 235.26 ± 1.05 mg/kg, and 155.60 ± 1.76 mg/kg, respectively. This is in agreement with the report of Wang, Ma [40] that describe in Chinese royal jelly, the most abundant elements were K, Mg, Ca, and Na while Fe, Zn, and Mn constituted were 17.78 ± 0.39, 24.79 ± 1.68 and 1.67 ± 0.06 mg/kg, respectively and less than K, Mg, Ca, and Na in royal jelly harvested at 3 days. Bengü, Ayna [41] reported that the current investigation is supported by the fact that K, Mg, Ca, and Na are lower than the total estimated components in royal jelly collected at various intervals following grafting. This showed that, harvesting times have an impact on the differentiating the macro and trace element contents in royal jelly harvested on 3^rd^ and 6^th^ days. These trace elements’ biological activities are essential to the biological properties of royal jelly [5].

### B-vitamins profile

The chromatogram obtained in analyzing a mixt of B- vitamin standards in a royal jelly sample (Vitamin B2, vitamin B3, vitamin B6 and vitamin B9) was shown in Fig 1. Each identified peak gave the different absorbance spectrum as that in the analytical standard, giving confidence in the confirmation of analytes (S2 Table in S1 File).

**Fig 2:**
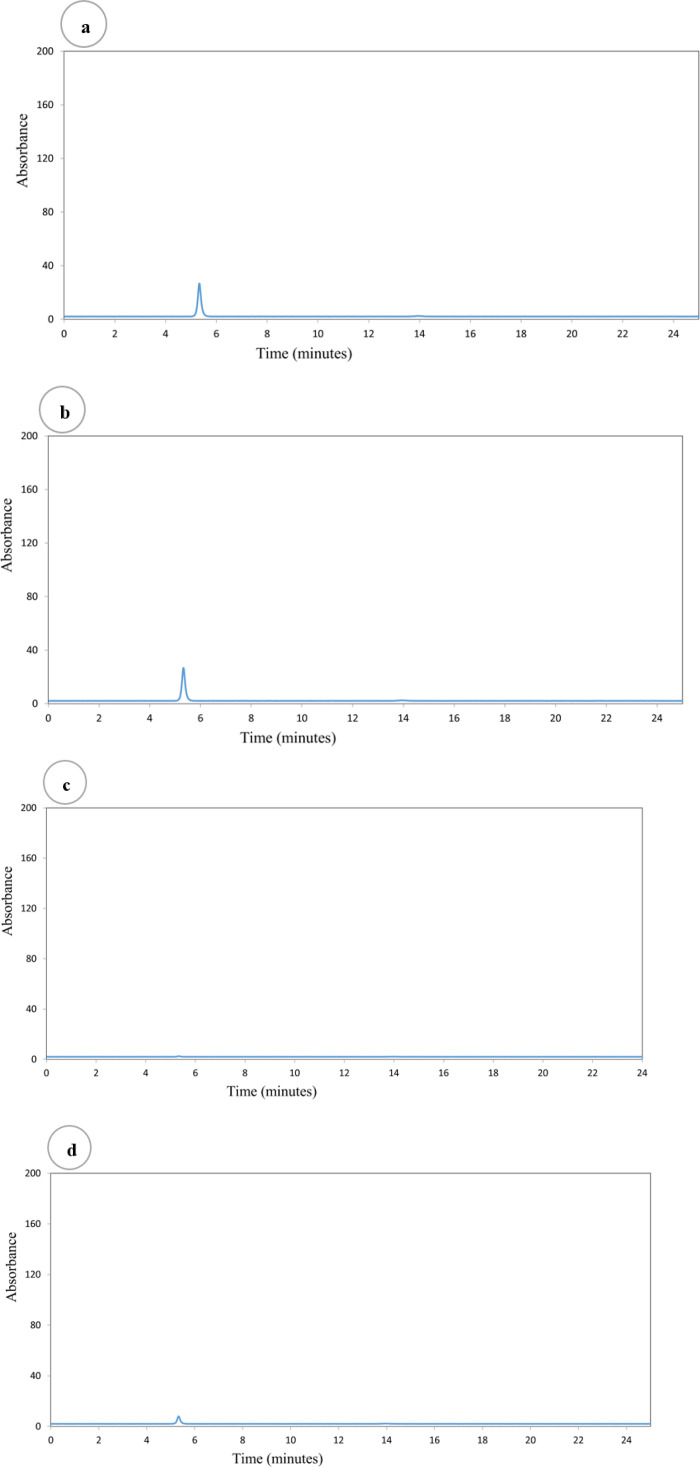
HPLC chromatograms of B-vitamin profiles in royal jelly: vitamin B2(a), vitamin B3 (b), vitamin B6 (c) and vitamin B9 (d).

The HPLC chromatograms of B-vitamin are presented in Fig. 2. The mean ± SD of B-vitamins profile for royal jelly was presented in Table 3. The results of B-vitamins profile showed that Vitamin B2 (2.03 ± 0.03 mg/kg), (1.76 ± 0.06) mg/kg; Vitamin B3 (1.15 ± 0.14 mg/kg), (0.57 ± 0.06) mg/kg; Vitamin B6 (1.5 ± 0.07 mg/kg), (0.9 ± 0.07 mg/kg) and Vitamin B9 (14.8 ± 0.07 mg/kg), (12.3 ± 0.35 mg/kg) of the royal jelly harvested at 3^rd^ and 6^th^ days respectively and significant (ρ < 0.05). Like for vitamin B concentrations (S18-21 Tables in S1 File), there was a significant difference in DPPH scavenging activity (S10 Table in S1 File) royal jelly samples (Table 3). The highest amount of vitamin B contents presented harvested at 3^rd^ day as compared to 6^th^ days. As shown in Table 3, the highest content was vitamin B9 (14.8 ± 0.07 mg/kg) and the lowest was vitamin B3 (0.57 ± 0.06 mg/kg) harvested at 3^rd^ and 6^th^ days respectively. These results are in line with those reported by Bărnuţiu, Mărghitaș [42]. However, the most abundant vitamins in royal jelly are niacin (B3) and pantothenic acid (B5), which are 52.8 and 42.42 mg/kg, respectively. These results are disagreed with those reported by Bărnuţiu, Mărghitaș [42] and Kamyab, Gharachorloo [43] and the results of vitamin B9 was 0.40 mg/kg and low as compared to the current study. However, the most abundant vitamins in royal jelly are niacin (B3) and pantothenic acid (B5), which are 52.8 and 42.42 mg/kg, respectively [42].

**Table 3:** B- vitamins: riboflavin (B2), niacin (B3), pyridoxine (B6), and folate (B9) in royal jelly collected on the 3^rd^ and 6^th^ days (mg/kg) and DPPH scavenging activity (%).

Treatments	Vitamin B2	vitamin B3	Vitamin B6	vitamin B9	DPPH %
Royal jelly 3^rd^ day	2.03 ± 0.03^a^	1.15 ± 0.14^a^	1.5 ± 0.07^a^	14.8 ± 0.07^a^	75.38 ± 1.95^a^
Royal jelly 6^th^ day	1.76 ± 0.06^b^	0.57 ± 0.06^b^	0.9 ± 0.07^b^	12.3 ± 0.35^b^	59.97 ± 3.49^b^

DPPH = 1, 1-diphenyl-2-picrylhydrazyl scavenging activity; SD-standard deviation; values show the average of duplicates ± standard deviation; Mean values in the same column with different letters are statistically significant different (ρ < 0.05).

Hydrosoluble vitamins B2, B3, B6, and B9 are vital chemical compounds that serve as coenzymes in a number of metabolic processes. Four B complex vitamins were identified in royal jelly samples harvested at 3^rd^ and 6^th^ days, as part of the current study. The findings demonstrated that higher quantities of B-vitamins were generally observed when collected early on three days. This may vary somewhat depending on the bees’ diet and storage factors. Fresh royal jelly that has been harvested early is the best for its nutritional value and this was agreement with the findings of Emir [44].

### 2,2-Diphenyl-1-picrylhydrazy (DPPH) scavenging activity of royal jelly

The DPPH assay measures the royal jelly sample’s capacity to donate hydrogen to the DPPH radical, which results in quantitative and the results was presented as shown in Table 3, examined the royal jelly’s antioxidant properties as expressed as radicals-scavenge effect upon DPPH (%), (75.38 ± 1.95) and (59.97 ± 3.49) harvested at 3^rd^ and 6^th^ days respectively (ρ < 0.05). The obtained results were compared depending on the harvested time (3^rd^ and 6^th^ days). Royal jelly’s strongest scavenging effect was consistently observed in samples obtained from the youngest larvae (three days old). Moreover, royal jelly collected at 72 hours after the larval transfer of the three day old larvae showed the greatest DPPH radical-scavenging activity among royal jelly samples collected at 6^th^ days (ρ < 0.05). These results are in line with those reported by Kocot, Kiełczykowska [17] and that the samples obtained from the youngest larvae (1 day old) placed in bee hives for the shortest period of time (24 hours) consistently showed the strongest scavenging effect of royal jelly. But, this was contradicted with the report of Liu, Yang [19] regardless of the original larval age, royal jelly collected 24 hours after the larval transfer showed a considerably higher DPPH radical-scavenging activity than those collected 48 or 72 hours after the larval transfer, Hence, the authors suggest that royal jelly’s antioxidant compounds and, thus, its therapeutic potential, may be affected by the time of harvest [17,45]. Accordingly, it is suggested that the overall polyphenolic levels would drop as the royal jelly was harvested more than 72 hours after the larval transfer, which would lower the antioxidant activities [19,46]. In addition to harvesting times of royal jelly, Vitamins aid in reducing oxidative stress and neutralizing free radicals [47]. Vitamin B9 can be an indirect indicator of the free radical scavenging activity in royal jelly, which was in agreement with the report of Joshi, Adhikari [48]. Vitamin B9 helps to reduce oxidative stress by lowering homocysteine, boosting glutathione, and preserving DNA, even though it is not a main antioxidant [49]. Folate has also been reported by several studies to be beneficial in improving hematological disease, cancer, neurological disorders, and neural tube defects [50]. The protective role of folates in these diseases was suggested to be due to its antioxidant property and folic acid(vitamin B9) is an effective free radical scavenger [48,51]. According to Verhaar, Stroes [52], vitamin B9 has significant antioxidant activities because of their potential to improve cardiovascular disease due to the ability to reduce plasma homocysteine level. This is based on the principle that harvesting time can all affect the precise concentrations and relationships between these vitamins. According to previous studies, royal jelly has strong antioxidant activity that protects against reactive oxygen species like superoxide anion and hydroxyl radicals. It has been suggested that the vitamins, polyphenolic compounds, or protein fractions may be partially responsible for this antioxidant activity [19, 46, 53–]. Harvesting time has an impact on royal jelly’s antioxidant capacity, notably its vitamin B content. Fresh harvested royal jelly typically exhibits the highest antioxidant capacity [19,54]. Therefore, the time of harvest affected the antioxidant potencies in royal jelly, and royal jelly harvested at 3^rd^ days showed the most substantial antioxidant activities.

### Determination of DPPH inhibition activity (IC_50_) of royal jelly

**Fig 3:**
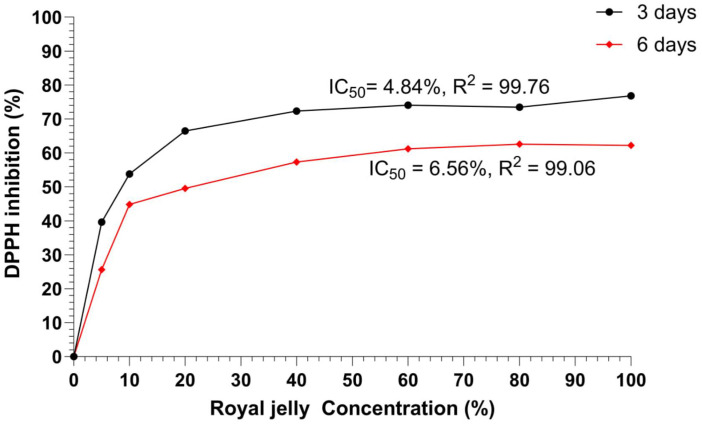
DPPH inhibition activity (IC50) of royal jelly collected on 3rd and 6th day (values were described as % (µg/mL)).

Fig 3. shows the free radical scavenging activity of royal jelly samples collected on third and sixth day. Among the royal jelly samples, the third day showed the highest activity. This is in lined with Liu, Yang [19] and the time of harvest and the initial larval age did affect the antioxidant potencies in royal jelly, and royal jelly collected early after the larval transfer showed the most substantial antioxidant activities. The IC_50_ value of royal jelly collected on third and sixth was 4.84% and 6.56%, respectively (Fig 3).

Generally, the result of the low number of IC_50_ values (%) indicates a high level of antioxidant potential and vice versa. Results obtained in this study indicated that IC_50_ values of the DPPH assay ranged from 4.84% to 6.56% for royal jelly collected from 3^rd^ and 6^th^ day respectively (Fig 3). These findings are in agreement with those has mentioned by Emir [44], showing free radical scavenging activity 5.72% for royal jelly samples. On the contrary, Phonmat, Charoenphun [55] found the higher free radical scavenging activity of royal jelly samples with the means of 24.23% and 37.23%, respectively. The antioxidant capabilities of royal jelly are generally influenced by the time of harvesting and the early collected royal jelly have strong antioxidant potentials. Emir [44] and Balkanska, Marghitas [56] stated that antioxidant activity might also result from the presence of other antioxidant secondary metabolites in royal jelly and the antioxidant activity of phenolic compounds is mainly due to their redox properties, which allow them to act as reducing agents, hydrogen donors and singlet oxygen quenchers. These findings have demonstrated that differences seem to exist in the antioxidant potencies of RJ harvested at different times of harvest. Liu, Yang [19] suggested that as the RJ harvested later than 24 hours after the larval transfer, the total polyphenolic contents would decrease, leading to a reduction in the antioxidant activities.

## Conclusion

Royal jelly is a remarkable honeybee product that is rich in nutrients and possesses a variety of biological properties. This study reveals the sugar, mineral, B-vitamin profiles and antioxidant properties of royal jelly collected at different harvesting times. Fructose, glucose, sucrose, and maltose sugars were found in every sample of royal jelly. These sugars are relatively low in royal jelly compared to other bee products like honey, making it less sweet. The concentrations of mineral and B- vitamin components in royal jelly varied considerably over harvesting time in the current investigation. It can be used as a nutritious and healthful food supplement for humans.

Compared to samples collected on the six days, royal jelly samples collected on three days consistently showed the strongest scavenging effect. In conclusion, in this study, the results showed that royal jelly collected on the third day has a good quality sugars, minerals, and B vitamins and showed the most substantial antioxidant activities. The present study also demonstrated that royal jelly has strong potential for antioxidative activities, suggesting harvesting time affects antioxidants, its effectiveness for managing oxidative stress, and its various beneficial impacts on the human body. Therefore, the result of the study showed that the harvesting time of royal jelly had an effect on the sugars, minerals, vitamin B and antioxidant properties and earlier harvested royal jelly is more nutritious. An in vivo study is needed to explore potential biological properties of royal jelly for further investigation. It is also advised that more research be done to determine the other vitamins in royal jelly.

## Supporting information

S1 File S1 Fig. HPLC Chromatogram of Sugar standard (where retention time of Fructose = 9.517, Glucose = 10.676, Sucrose = 13.742, and Maltose = 14.799). **S2 Fig.** Calibration graph of glucose in g/100g. **S3 Fig.** Calibration graph of fructose in g/100g. **S4 Fig**. Calibration graph of sucrose in g/100g. **S5 Fig.** Calibration graph of maltose in g/100g. **S6 Fig.** Calibration graph of Na in ppm. **S7 Fig**. Calibration graph of K in ppm. **S8 Fig**. Calibration graph of Mg in ppm. **S9 Fig.** Calibration graph of Ca in ppm. **S10 Fig**. Calibration graph of Fe in ppm. **S11 Fig.** Calibration graph of Zn in ppm. **S12 Fig.** Calibration graph of Mn in ppm. **S1 Table**. Sugar profile of Royal Jelly (g/100g). **S2 Table.** Vitamins profiles of Royal Jelly (mg/100g). **S3 Table**. Mineral profiles of Royal Jelly (ppm). **S4 Table**. % DPPH scavenging activities of Royal Jelly. **S5 Table**. Raw data for DPPH inhibition activity (IC50) of royal jelly. **S6 Table.** ANOVA table for fructose. **S7 Table.** ANOVA table for glucose. **S8 Table.** ANOVA table for sucrose content. **S9 Table**. ANOVA table for maltose content. **S10 Table**. ANOVA table for DPPH. **S11 Table**. ANOVA table for Ca. **S12 Table.** ANOVA table for Na. **S13 Table**. ANOVA table for K. **S14 Table**. ANOVA table for Fe. **S15 Table**. ANOVA table for Zn. **S16 Table.** ANOVA table for Mn. **S17 Table.** ANOVA table for Mg. **S18 Table**. ANOVA table for Vitamin B2. **S19 Table.** ANOVA table for Vitamin B3. **S20 Table**. ANOVA table for Vitamin B6. **S21 Table.** ANOVA table for Vitamin B9.
(ZIP)
