# Supplementary material for: Sugar, mineral, B-vitamins profiles and radical scavenging activity of Royal jelly collected at different harvesting times
Source: PLoS One. 2026 Apr 21;21(4):e0347342. doi: 10.1371/journal.pone.0347342 (PMC13098920; doi:10.1371/journal.pone.0347342)
Supplement: S1 File — HPLC Chromatogram of Sugar standard (where retention time of Fructose = 9.517, Glucose = 10.676, Sucrose = 13.742, and Maltose = 14.799). S2 Fig. Calibration graph of glucose in g/100g. S3 Fig. Calibration graph of fructose in g/100g. S4 Fig. Calibration graph of sucrose in g/100g. S5 Fig. Calibration graph of maltose in g/100g. S6 Fig. Calibration graph of Na in ppm. S7 Fig. Calibration graph of K in ppm. S8 Fig. Calibration graph of Mg in ppm. S9 Fig. Calibration graph of Ca in ppm. S10 Fig. Calibration graph of Fe in ppm. S11 Fig. Calibration graph of Zn in ppm. S12 Fig. Calibration graph of Mn in ppm. S1 Table. Sugar profile of Royal Jelly (g/100g). S2 Table. Vitamins profiles of Royal Jelly (mg/100g). S3 Table. Mineral profiles of Royal Jelly (ppm). S4 Table. % DPPH scavenging activities of Royal Jelly. S5 Table. Raw data for DPPH inhibition activity (IC50) of royal jelly. S6 Table. ANOVA table for fructose. S7 Table. ANOVA table for glucose. S8 Table. ANOVA table for sucrose content. S9 Table. ANOVA table for maltose content. S10 Table. ANOVA table for DPPH. S11 Table. ANOVA table for Ca. S12 Table. ANOVA table for Na. S13 Table. ANOVA table for K. S14 Table. ANOVA table for Fe. S15 Table. ANOVA table for Zn. S16 Table. ANOVA table for Mn. S17 Table. ANOVA table for Mg. S18 Table. ANOVA table for Vitamin B2. S19 Table. ANOVA table for Vitamin B3. S20 Table. ANOVA table for Vitamin B6. S21 Table. ANOVA table for Vitamin B9. (ZIP) [file pone.0347342.s001.zip › Supporting Information/S10 Table.docx]

**S10 Table. ANOVA table for DPPH**

| Source | DF | Sum of Squares | Mean Square | F Value | Pr > F |
| --- | --- | --- | --- | --- | --- |
| Model | 1 | 356.12 | 356.121 | 44.58 | 0.003 |
| Error | 4 | 31.95 | 7.988 |  |  |
| Total | 5 | 388.07 |  |  |  |
